# Establishment of a Sensitive qPCR Methodology for Detection of the Olive-Infecting Viruses in Portuguese and Tunisian Orchards

**DOI:** 10.3389/fpls.2019.00694

**Published:** 2019-05-29

**Authors:** Maria Doroteia Campos, Mohamed Salem Zellama, Carla Varanda, Patrick Materatski, Augusto Peixe, Maher Chaouachi, Maria do Rosário Félix

**Affiliations:** ^1^ICAAM – Instituto de Ciências Agrárias e Ambientais Mediterrânicas, Instituto de Investigação e Formação Avançada, Universidade de Évora, Évora, Portugal; ^2^Laboratoire de Recherche “Bioressources: Biologie Intégrative & Valorisation," Institut Supérieur de Biotechnologie de Monastir, Université de Monastir, Monastir, Tunisia; ^3^Departamento de Fitotecnia, ICAAM – Instituto de Ciências Agrárias e Ambientais Mediterrânicas, Escola de Ciências e Tecnologia, Universidade de Évora, Évora, Portugal

**Keywords:** *OLEA europaea*, qPCR, viral diseases, virus detection, sensitive detection

## Abstract

Sensitive detection of viruses in olive orchards is actually of main importance since these pathogenic agents cannot be treated, their dissemination is quite easy, and they can have eventual negative effects on olive oil quality. The work presented here describes the development and application of a new SYBR^®^ Green-based real-time quantitative PCR (qPCR) analysis for specific and reliable quantification of highly spread olive tree viruses: *Olive latent virus 1* (OLV-1)*, Tobacco necrosis virus D* (TNV-D), *Olive mild mosaic virus* (OMMV), and *Olive leaf yellowing-associated virus* (OLYaV). qPCR methodology revealed high specificity and sensitivity, estimated in the range of 0.8–8 copies of the virus genome, for the studied viruses. For validation of the method, total RNA and double strand RNA (dsRNA) from naturally infected trees were used. In a first trial, dsRNAs from trees of cv. “Galega vulgar” from a Portuguese orchard, were subjected to qPCR and from the 30 samples tested, 26 were TNV-D and/or OMMV-positive and 25 were OLV-1 positive. In a second trial, total RNA from trees of different cultivars from Tunisian orchards, were here tested by qPCR and all viruses were detected. From the 33 samples studied, the most prevalent virus detected in Tunisia orchards was OLV-1 (31 samples diagnosed), followed by OLYaV (20 samples diagnosed), and finally the combination in last TNV-D and/or OMMV (12 samples diagnosed). In both trials, qPCR demonstrated to be effective and sensitive, even when using total RNA as template. qPCR through the use of a SYBR^®^ Green methodology enabled, for the first time, a reliable, sensitive, and reproducible estimation of virus accumulation in infected olive trees, in which viruses are usually in low titres, that will allow gaining new insights in virus biology essential for disease control and give an important contribution for establishment of sanitary certification of olive propagative material.

## Introduction

Olive trees are susceptible to several pathogens that may affect the yield and quality of their products with important economic impact, such as diseases causes by fungi (i.e., *Colletotrichum* spp. and *Spilocaea oleagina*) (Salman, 2017; Materatski et al., 2018), and more recently the emergence of *Xylella fastidiosa,* first noticed in 2013, and responsible for a severe outbreak of Olive quick decline syndrome (Saponari et al., 2013). Regarding virus infection, plants infected usually present morphological and physiological alterations, which always incurs in inferior performance such as the decreased host biomass and crop yield loss, with chloroplast known to be highly involved in those processes (Zhao et al., 2016). Olive trees are affected by several viruses, with 15 olive viruses identified until now (Félix et al., 2012). Viral symptoms on olive trees include bumpy fruits, leaf yellowing, vein banding, and vein clearing, (Martelli, 1999; Albanese et al., 2012). These symptoms are non-specific and difficult to recognize, and often occur in virus-infected olive trees without apparent symptoms, which makes viral diagnosis in the field impossible to perform (Martelli et al., 2002; Alabdullah et al., 2010; Martelli, 2011; Zellama et al., 2018). A decrease in oil yield and maturity index in virus infected olives was also reported, as well as elevated total phenols in olive oil content from infected olives when compared to healthy fruits (Godena et al., 2012). Nevertheless, the *Olive leaf yellowing associated virus* (OLYaV) was not found to have a negative interference in oil yield and quality (Fontana et al., 2019).

Virus surveys carried in Mediterranean countries and others, concerning eight olive-infecting viruses (SLRSV, *Strawberry latent ringspot virus*; ArMV, *Arabis mosaic virus*; CLRV, *Cherry leafroll virus*; OLRSV, *Olive latent ringspot virus*; CMV, *Cucumber mosaic virus*; OLYaV, *Olive leaf yellowing-associated virus*; OLV-1, *Olive latent virus 1*; OLV-2, *Olive latent virus 2*), have shown high levels of infection, such as 75% in Tunisia, 51% in Syria, 33% in Italy, 31% in Lebanon, 25% in Croatia, and 31% of necroviruses infection in Portugal (Al Abdullah et al., 2005; Fadel et al., 2005; Faggioli et al., 2005; Varanda et al., 2010; Luigi et al., 2011; Zellama et al., 2018). However, the high presence of double stranded RNA (dsRNA) in olive orchards suggests even higher levels of infection (Félix et al., 2002; Saponari et al., 2002) that may be identified with new and more sensitive diagnostic techniques.

Phytosanitary certification programs depend on robust diagnostic procedures for detection of the olive viruses, since their spread will mainly depend on preventive measures such as the use of pathogen-tested propagative material (Albanese et al., 2012). Following the EU regulation on the marketing of fruit plants intended for fruit production, namely the Commission Directive 93/48/EEC (EEC Directive, 1993), the *Conformitas Agraria Communitatis* (*CAC*) category, applicable to olive plant material, demands that plants must be free of all viruses (Annex to the 93/48/EEC). The Italian certification of propagative material (DM 20/11/2006), followed by most of the Mediterranean countries, including the countries from the North of Africa, imposes the absence of several viruses such as ArMV, CLRV, SLRSV, CMV, OLV-1, OLV-2, OLYaV, and *Tobacco necrosis virus senso lato*, in which *Tobacco necrosis virus* D (TNV-D) and *Olive mild mosaic virus* (OMMV) are included (Albanese et al., 2012). The adoption of such certification programs requires the use of extremely sensitive diagnostic techniques for viral detection, which are the basis for valid programs. The low viral titres in olive tissues are the major constraint of the techniques, and do not always allow the successful, accurate and reproducible detection (Zellama et al., 2018).

Traditionally, unreliability techniques based on serology tests (ELISA) have been used for the detection of viruses (Martelli, 1999; Bertolini et al., 2001). More recently, molecular biology-based methods that include the highly laborious dsRNA analysis have been performed, once dsRNA reduces the constraint caused by the common low viral concentration in trees (Varanda et al., 2010, 2014; Zellama et al., 2018). RT-PCR has demonstrated to be the most rapid, sensitive, and reliable technique (Varanda et al., 2010; Luigi et al., 2011; Albanese et al., 2012; Zellama et al., 2018), using different templates as viral targets such as TNA, total RNA or dsRNA. The advancements of the molecular virology and biotechnology have witnessed major breakthroughs in the recent years resulting in highly sensitive and effective technologies/methods (Yadav and Khurana, 2016). In plant virology, real-time quantitative PCR (qPCR) is increasingly being used to improve sensitivity and accuracy while maintaining reliability (Poojari et al., 2016; Malandraki et al., 2017). The use of qPCR instrumentation presents several advantages, since it requires considerably short hands-on time, and detection of amplified products is automated, simple, and reproducible (Espy et al., 2006).

In the work described here, three new SYBR^®^ Green qPCR assays were developed for reliable and sensitive detection of OLV-1, TNV-D and/or OMMV, and OLYaV. The work involved the use of specific primers for each target virus, using different templates (total RNA and dsRNA). The main performance criteria were tested such as the sensitivity of the technique and the specificity of the primers. qPCR was validated through application to plant material from different olive orchards, from Portugal and Tunisia.

## Materials and Methods

### Virus Isolates and Plant Material

Virus reference isolates of OLV-1, TNV-D, and OMMV were obtained according to Varanda et al. (2014). For OLYaV, since this virus is not mechanically transmitted (van Regenmortel et al., 2000), a sequenced cDNA obtained from an infected olive tree was used as reference (Zellama et al., 2018).

For validation and comparative analysis of virus infection, two different experiments were conducted. In the first one, samples were collected from 30 symptomatic or asymptomatic olive trees from an orchard under traditional management. The cultivar studied was the native cultivar “Galega vulgar,” and the orchard was located in Alentejo region (south Portugal).

The second experiment was performed in samples collected from a total of 33 symptomatic or asymptomatic olive trees. These samples were randomly selected from the 280 collected by Zellama et al. (2018), but with the care to select 3 trees per each orchard. The sampled trees belonged to 11 orchards, under different types of management: three traditional, six semi-intensive, and two intensive. The orchards were located in several regions covering whole Tunisia. Sampled trees belonged to the Tunisian native cultivars “Chemlali,” “Chétoui,” “Meski,” and to the introduced cultivars “Picholine,” “Koroneiki,” and “Arbequina.” All samples consisted of four cuttings of ca. 20 cm in length collected from 2-year stems from each quadrant of the canopy of each tree, that were kept in plastic bags at 4°C, until further use.

### Nucleic Acids Extraction and Reverse Transcription

In the first experiment, double stranded RNAs (dsRNAs) were extracted from 300 mg of cortical scrapings according to Morris and Dodds (1979). dsRNAs were visualized by 0.8% agarose gel electrophoresis prior to denaturation by heating at 100°C for 5 min, followed by 15 min on ice. cDNA was produced by RevertAid H Minus Reverse Transcriptase (Thermo Scientific), according to manufacturer’s instructions, with random hexamers (Promega).

In the second experiment, total RNA was extracted from 25 mg of cortical scrapings from cuttings that were mixed together and homogenized using liquid nitrogen. Total RNA was extracted using the RNeasy Plant Mini Kit (Qiagen), following the manufacturer’s instructions. The quantification of RNA and the evaluation of its purity were determined in a NanoDrop-2000C spectrophotometer (Thermo Scientific). RNA integrity was evaluated by denaturing gel electrophoresis. Total RNA (500 ng) was reverse transcribed with the Maxima First Strand cDNA Synthesis Kit (Thermo Scientific), according to manufacturer’s instructions.

### Conventional PCR Assays

For conventional PCR assays, primers used for OLV-1 and OLYaV were the same as in Zellama et al. (2018) (Table 1). For the detection of OMMV and TNV-D, a single set of primers that allow the amplification of both TNV-D and OMMV was used (Table 1; Cardoso et al., 2004). 2 μl of cDNA were used in PCR carried out in 1x DreamTaq Buffer (Thermo Scientific), 0.2 mM dNTPs, 0.5 μM of each primer and 2.5 U of DreamTaq DNA Polymerase (Thermo Scientific) in a total volume of 50 μl. Amplifications were carried out in a Thermal Cycler (Bio-Rad) at 95°C for 1 min, 35 cycles at 95°C for 30 s, 54°C (for OLV1 and both TNV-D and OMMV) or 58°C (for OLYaV) for 1 min, 72°C for 1 min, and a final extension step of 72°C for 10 min. Amplified products were analyzed by electrophoresis in 1% agarose gel.

**Table 1:** Primers used on conventional PCR assays.

Species	Primers (5′ → 3′)	AS (bp)
OMMV and/or TNV-D	Fw: GTGTTCAGTCATATACATACC	247
	Rv: GCCTATTGTGCTGTACCAC	
OLV-1	Fw: TTTCACCCCACCAAATGGC	747
	Rv: CTCACCCATCGTTGTGTGG	
OLYaV	Fw: CGAAGAGAGCGGCTGAAGGCTC	346
	Rv: GGGACGGTTACGGTCGAGAGG	


*Description of forward (Fw) and reverse (Rv) primers used for specific detection of *Olive mild mosaic virus* (OMMV) and/or *Tobacco necrosis virus* D (TNV-D) (Cardoso et al., 2004), *Olive latent virus 1* (OLV-1) and *Olive leaf yellowing-associated virus* (OLYaV) in the conventional PCR assays (Zellama et al., 2018). AS, amplicon size.*

### SYBR^®^ Green qPCR Assays

These assays were performed in both experiments. For TNV-D and/or OMMV it was used the already referred primer set (Cardoso et al., 2004). For OLV-1 a gene-specific primer set was designed in a conserved region of the capsid protein (CP), after alignment of all full-genome and CP sequences collected from NCBI GenBank database (accession n° KF804054). For OLYaV the same procedure was followed, but the primers were designed in the conserved gene coding the heat shock protein 90 (accession n° AJ844555). The Primer Express 3.0 Software (Applied Biosystems) was used for the design of the primers, using the default parameters of the software, and their specificity was tested *in silico* using basic local alignment search tool (BLAST) at the National Center for Biotechnology Information (NCBI)^1^. All qPCR primers are listed in Table 2.

**Table 2:** Primers used on qPCR assays.

Species	Accession ID	Primers (5′ → 3′)	AS (bp)	References
OMMV and/or TNV-D	AY616760	Fw: GTGTTCAGTCATATACATACC	247	Cardoso et al. (2004)
		Rv: GCCTATTGTGCTGTACCAC		
OLV-1	KF804054	Fw: GGGGTATGATGGTGCTATGG	162	This work
		Rv: ACTCCGCAATATCCGTTCTG		
OLYaV	AJ844555	Fw: GCTTATCTACTACGCCGATCTTGTC	71	This work
		Rv-AAGAGTGGATCCATCTAGATCGAAA		


*Description of forward (Fw) and reverse (Rv) primers used for specific detection of *Olive mild mosaic virus* (OMMV) and/or *Tobacco necrosis virus D* (TNV-D), *Olive latent virus 1* (OLV-1) and *Olive leaf yellowing-associated virus* (OLYaV), in the qPCR assays. AS, amplicon size.*

qPCRs were carried out on a 7500 Real Time PCR System (Applied Biosystems) with SYBR Green q-PCR Master Mix (Nzytech). 5 μL of first-strand cDNA (previously diluted 1:10) and 560 nM of each specific primer were used, performing in a total 18 μl reaction volume. The quantification cycle (Cq) values were acquired for each sample with the following cycling conditions: 10 min at 95°C for initial denaturation, an amplification program of 40 cycles at 95°C for 15 s and 60°C for 1 min. Three technical replicates were considered for each sample. Virus reference isolates and no template controls were included in all plates. The fluorescence threshold was manually set above the background level. The specificity of qPCR reactions was evaluated by melting curve analysis. The identity of each amplicon was confirmed by Sanger sequencing. Specific amplification of target viral cDNA was confirmed by cloning PCR amplicons into pGem^®^-T Easy vector (Promega) and used to transform *Escherichia* *coli* JM109 (Promega) competent cells, by standard methodologies. Each amplicon, sequenced through Sanger procedure, was identified by comparison with corresponding virus sequences available from GenBank.

The specificity of each SYBR^®^ Green assay against the cDNA of the other virus reference isolates was also tested by qPCR.

For each specific viral fragment, to determine the amplification efficiencies of the primers, standard curves were generated from 10-fold dilution series of plasmid DNA (each viral amplicon were cloned into a plasmid vector, as described above), used to draw a nine-point calibration curve to validate each assay in the dynamic range chosen (0.8 to 8E7 target copies). Amplification efficiencies were calculated through the equation E = (10^(-1/slope)^ - 1) × 100, as well as slope and linearity (coefficient of determination, R^2^). Detection sensitivities of the assays were determined from the Cq of the lowest plasmid dilution that fell within the linear standard curve. The method performed for absolute DNA quantification was based on the determination of the absolute number of target copies (TCN) previously described by Campos et al. (2018).

## Results

### Linearity, Sensitivity, and Specificity of qPCR Assays

**FIGURE 1:**
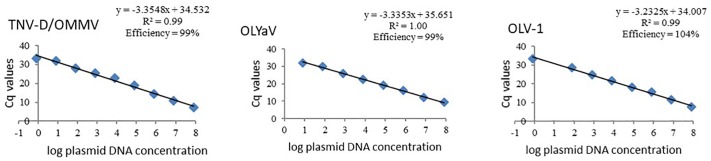
Standard curves of olive viruses constructed based on Cq values obtained from a ten-fold dilution series of each target plasmid DNA in the dynamic range of 0.8 to 8E7 target copies. TNV-D, *Tobacco necrosis virus*; OMMV, *Olive mild mosaic virus*; OLV-1, *Olive latent virus 1*; OLYaV, *Olive leaf yellowing-associated virus*; R^2^, regression coefficients.

Standard curves of all studied olive viruses were constructed based on Cq values obtained from a 10-fold dilution series of the target plasmid DNA obtained from each reference isolate, in the dynamic range that was chosen. Linear relationships between Cq values and *log* plasmid DNA were obtained for all viruses, with regression coefficients (R^2^) above 0.99 (Figure 1). As expected, similar standard curves were obtained when OMMV and TNV-D were used as targets, once a single set of primers for amplification of both viruses was used. The efficiencies (E) for all virus-specific primer pairs ranged from 99% to 104% (Figure 1).

The detection sensitivity of each assay was determined from the Cq of the lowest plasmid dilution that fell with standard curve, that enable to detect 8 target copies of OLYaV and 0.8 target copies of TNV-D/OMMV and OLV-1 (Table 3). The Cq values that correspond to the detected target copies are indicated in Table 3.

**Table 3:** Detection limits of each of the three qPCR assays based on determination of copy numbers.

Virus	Cq value	Copy number
TNV-D/OMMV	33.27	0.8
OLV-1	33.36	0.8
OLYaV	32.07	8


*The detection range was determined from standard curves generated from the dilution series of respective recombinant plasmid DNA. The mean Cq values of positive samples were represented with respective copy numbers. TNV-D, *Tobacco necrosis virus D*; OMMV, *Olive mild mosaic virus*; OLV-1, *Olive latent virus 1*; OLYaV, *Olive leaf yellowing-associated virus*.*

The specificity of the assays was confirmed *in silico*, performing homology search similarities against NCBI databases, and experimentally, with each reference virus isolates targeted only by the respective SYBR^®^ Green assay (not shown). Also, the specificity of the primers and their combinations were verified via cloning and sequencing of amplified products.

### Validation of Real-Time qPCR Assays Using Field Samples

To evaluate the practical robustness and accuracy of the qPCR assays, two experiments were conducted to test the presence of the viruses using field samples. Additionally, conventional PCR assays were also performed. It was also studied if total RNA template as viral target was suitable for virus detection through qPCR.

In the first experiment, concerning the use of dsRNA from 30 field-collected samples from a Portuguese olive orchard, OLYaV was not detected with neither qPCR nor conventional PCR. For the other studied viruses, qPCR revealed 26 positive samples for TNV-D and/or OMMV and 25 positive samples for OLV-1 (Table 4 and Supplementary Table 1). Using conventional PCR, only six samples were positive for TNV-D and/or OMMV and three samples were positive to OLV-1.

**Table 4:** Comparison of SYBR^®^ Green-based real-time quantitative reverse transcription PCR assays with conventional PCR, for virus detection in field-collected samples from a Portuguese olive orchard, with cv. “Galega vulgar.”

Virus	SYBR Green-based PCR (qPCR) (Virus-positive/total number of samples)	Conventional PCR (RT-PCR) (Virus-positive/total number of samples)
TNV-D and/or OMMV	26/30	6/30
OLV-1	25/30	3/30
OLYaV	0/30	0/30


*TNV-D, *Tobacco necrosis virus D*; OMMV, *Olive mild mosaic vírus*; OLV-1, *Olive latent virus 1*; OLYaV, *Olive leaf yellowing-associated virus*.*

To demonstrate the suitability of the qPCR methodology in different olive material and corroborate qPCR sensitivity, a second experiment was performed in 33 field-collected samples from 11 olive orchards, in several regions of Tunisia. For the qPCR experiment it was used as template total RNA from cortical scrapings from cuttings, instead of the highly laborious protocol for extraction of dsRNA used for conventional PCR. The results revealed that through qPCR and using total RNA as template, it was possible to detect all the studied viruses (Table 5 and Supplementary Table 1). qPCR revealed that the most prevalent virus detected in Tunisia orchards was OLV-1 (31 samples diagnosed), followed by OLYaV (20 samples diagnosed), and finally the combination in last TNV-D and/or OMMV (12 samples diagnosed). Results on conventional PCR revealed that 11 samples were OLV-1 positive, 10 samples were TNV-D and/or OMMV-positive and 10 samples were OLYaV positive, as determined by Zellama et al. (2018).

**Table 5:** Comparison of SYBR^®^ Green-based real-time quantitative reverse transcription PCR assays with conventional PCR, for virus detection in field-collected samples from 11 olive orchards, in several regions of Tunisia.

Virus	SYBR Green-based PCR (qPCR) (Virus-positive/total number of samples)	Conventional PCR (RT-PCR) (Virus-positive/total number of samples)
TNV-D and/or OMMV	12/33	10/33
OLV-1	31/33	11/33
OLYaV	20/33	10/33


*Conventional PCR results included in Zellama et al. (2018). TNV-D, *Tobacco necrosis virus D*; OMMV, *Olive mild mosaic vírus*; OLV-1, *Olive latent virus 1*; OLYaV, *Olive leaf yellowing-associated virus*.*

Both experiments performed here demonstrate qPCR tests as suitable for detection of virus infecting olive.

## Discussion

Since there is no known source of genetic resistance to olive viruses, a key component of viral disease management is the propagation of virus-free plant materials. The main approach used to obtain, propagate and commercialize plants free from harmful pathogens is through phytosanitary selection and certification programs. The obtention of pathogen-free material from infected trees is possible to achieve through sanitation treatments such as heat therapy, meristem tip culture and micrografting, although the use of these methodologies for virus elimination in olive is still limited (Albanese et al., 2012). Thus, sensitive, reliable and cost-effective assays for the detection of olive viruses are critical and of major importance to guarantee a sustainable production of the orchards. The use of the highly sensitive qPCR arises as an extremely useful tool for studying various agents of infectious. In plant pathology, this technology is increasingly being used for studying various causal agents of plant diseases, including viruses (Poojari et al., 2016; Malandraki et al., 2017; Abdullah et al., 2018; Baaijens et al., 2018; Campos et al., 2019).

The work performed here, describes for the first time the use of a SYBR^®^ Green-based qPCR methodology for specific and reliable quantitative detection of highly spread olive viruses. Moreover, despite the low viral titres of the olive viruses (Martelli, 1999), qPCR turns possible the use of total RNA for the detection of viruses, instead of the highly laborious dsRNA extraction essential for RT-PCR (Varanda et al., 2010, 2014; Félix et al., 2012; Zellama et al., 2018). The primers used in the experiments successfully differentiated the target pathogens, and the results obtained show that qPCR is not only a faster method, but most importantly, a sensitive method for the detection and quantification of olive viruses directly from woody plant material. It was used a primer set that allow the simultaneous detection of TNV-D and OMMV (designed in the CP gene, see Cardoso et al., 2004), and newly designed primers targeting OLV-1 and OLYaV, that revealed E, R^2^ and slope consistent with the acceptance criteria (Broeders et al., 2014), confirming the accuracy and linear response of the assays over a wide range of dilutions, and suggesting absence of PCR inhibitors. These performance criteria further demonstrate that the developed assays, additionally to detection, constitute a reliable quantitative tool suitable for population or competition studies among the studied viruses in mixed infections in a variety of olive hosts. Conventional PCR conditions are different from qPCR conditions, with different optimized protocols and primers (only TNV-D and/or OMMV primer set was common to both methodologies), and consequently with not comparable results. Nevertheless, the idea of the higher sensitivity qPCR methodology, even with the low viral titres in olive tissues, is here reinforced. In the Portuguese cv. “Galega vulgar” the viruses OLV-1, TNV-D and/or OMMV were identified while OLYaV was not detected. OLV-1, TNV-D and/or OMMV were already identified in Portugal by RT-PCR methodology, although with lower rates (Cardoso et al., 2004, 2009; Varanda et al., 2010). In Tunisian orchards, independently of the cultivar, type of management or region, OLYaV, OLV-1, TNV-D and/or OMMV were detected, confirming previous RT-PCR results (Zellama et al., 2018), although qPCR registered a higher level of infection for the viruses under study. These results reinforce the idea of the sensitivity of the qPCR methodology, regardless of cultivar or region.

qPCR combined with the SYBR^®^ Green methodology enabled, for the first time, a high level of reliability, sensitivity and reproducibility regarding the estimation of virus accumulation in infected olive trees, being an efficient tool for the early diagnosis of the diseases, and for the improvement of the viral diagnosis. The results achieved will allow gaining new insights in virus biology, essential for disease control. Furthermore, this robust diagnostic procedure will give an important contribute for the establishment of phytosanitary certification programs of olive and high-quality productions.

## Data Availability

The datasets generated for this study are available on request to the corresponding author.

## Author Contributions

MDC and MRF: conceptualization. MDC: formal analysis. MDC and AP: funding acquisition. MDC, MZ, and CV: methodology. MDC, MZ, CV, and PM: resources. MRF and MC: supervision. MDC: writing, original draft preparation. MDC, MZ, CV, PM, AP, MC, and MRF: writing, review and editing.

## Conflict of Interest Statement

The authors declare that the research was conducted in the absence of any commercial or financial relationships that could be construed as a potential conflict of interest.

## Footnotes

1 http://www.ncbi.nlm.nih.gov/
